# It is not the time to abandon intraoperative frozen section in endometrioid adenocarcinoma: A large‐scale, multi‐center, and retrospective study

**DOI:** 10.1002/cam4.5643

**Published:** 2023-01-31

**Authors:** Xiaohang Yang, Jingjing Yin, Yu Fu, Yuanming Shen, Chuyao Zhang, Shuzhong Yao, Congjian Xu, Min Xia, Ge Lou, Jihong Liu, Bei Lin, Jianliu Wang, Weidong Zhao, Jieqing Zhang, Wenjun Cheng, Hongyan Guo, Ruixia Guo, Fengxia Xue, Xipeng Wang, Lili Han, Xiaomao Li, Ping Zhang, Jianguo Zhao, Wenting Li, Yingyu Dou, Zizhuo Wang, Jingbo Liu, Kezhen Li, Gang Chen, Chaoyang Sun, Beibei Wang, Xingsheng Yang

**Affiliations:** ^1^ Cancer Biology Research Center (Key Laboratory of the Ministry of Education), Tongji Hospital, Tongji Medical College, Huazhong University of Science and Technology Wuhan Hubei China; ^2^ Department of Gynecology and Obstetrics Tongji Hospital, Tongji Medical College, Huazhong University of Science and Technology Wuhan Hubei China; ^3^ Women's Hospital, School of Medicine, Zhejiang University Hangzhou Zhejiang China; ^4^ Department of Gynecologic Oncology Sun Yat‐sen University Cancer Center Guangzhou China; ^5^ Department of Obstetrics and Gynecology The First Affiliated Hospital of Sun Yat‐sen University Guangzhou China; ^6^ Department of Gynecology Obstetrics and Gynecology Hospital of Fudan University Shanghai China; ^7^ Department of Gynecology and Obstetrics The Affiliated Yantai Yuhuangding Hospital of Qingdao University Yantai Shandong China; ^8^ Department of Gynecology Oncology Harbin Medical University Cancer Hospital Harbin China; ^9^ Department of Obstetrics and Gynecology Shengjing Hospital Affiliated to China Medical University Shenyang Liaoning China; ^10^ Peking University People's Hospital Beijing China; ^11^ Division of Life Sciences and Medicine The First Affiliated Hospital of USTC, University of Science and Technology of China Hefei Anhui China; ^12^ Department of Gynecologic Oncology Guangxi Medical University Cancer Hospital Nanning Guangxi China; ^13^ The First Affiliated Hospital of Nanjing Medical University Nanjing Jiangsu China; ^14^ The Third Hospital of Peking University Beijing China; ^15^ Department of Gynecology and Obstetrics the First Affiliated Hospital of Zhengzhou University Zhengzhou China; ^16^ Department of Gynecology and Obstetrics Tianjin Medical University General Hospital Tianjin China; ^17^ Department of Gynecology and Obstetrics XinHua Hospital, Shanghai JiaoTong University School of Medicine Shanghai China; ^18^ Department of Gynecology People's Hospital of Xinjiang Uygur Autonomous Region Urumqi China; ^19^ Department of Gynecology and Obstetrics The Third Affiliated Hospital, Sun Yat‐sen University Guangzhou China; ^20^ Department of Gynecology The Second Hospital of Shandong University Jinan Shandong China; ^21^ Department of Gynecologic Oncology Tianjin Central Hospital of Gynecology and Obstetrics, Affiliated Hospital of Nankai University; Tianjin Clinical Research Center For Gynecology and Obstetrics; Branch of National Clinical Research Center For Gynecology and Obstetrics Tianjin China; ^22^ Department of Obstetrics and Gynecology, Qilu Hospital Cheeloo College of Medicine, Shandong University Jinan Shandong China

**Keywords:** endometrioid adenocarcinoma, high‐grade, intraoperative frozen section, myometrial invasion, retrospective studies

## Abstract

**Introduction:**

Stage IB (deep myometrial invasion) high‐grade endometrioid adenocarcinoma (EA), regardless of LVSI status, is classified into high‐intermediate risk groups, requiring surgical lymph node staging. Intraoperative frozen section (IFS) is commonly used, but its adequacy and reliability vary between reports. Hence, we determined the utility of IFS in identification of high‐risk factors, including deep myometrial invasion and high‐grade.

**Method:**

We retrospectively analyzed 9,985 cases operated with hysterectomy and diagnosed with FIGO stage I/II EA in postoperative paraffin section (PS) results at 30 Chinese hospitals from 2000 to 2019. We determined diagnostic performance of IFS and investigated whether the addition of IFS to preoperative biopsy and imaging could improve identification of high‐risk factors.

**Results:**

IFS and postoperative PS presented the highest concordance in assessing deep myometrial invasion (Kappa: 0.834), followed by intraoperative gross examination (IGE Kappa: 0.643), MRI (Kappa: 0.395), and CT (Kappa: 0.207). IFS and postoperative PS presented the highest concordance for high‐grade EA (Kappa: 0.585) compared to diagnostic curettage (D&C 0.226) and hysteroscope (Hys 0.180). Sensitivity and specificity for detecting deep myometrial invasion were 86.21 and 97.20% for IFS versus 51.72 and 88.81% for MRI, 68.97 and 94.41% for IGE. These figures for detecting high‐grade EA were 58.21 and 96.50% for IFS versus 16.42 and 98.83% for D&C, 13.43 and 98.64% for Hys. Parallel strategies, including MRI‐IFS (Kappa: 0.626), D&C‐IFS (Kappa: 0.595), and Hys‐IFS (Kappa: 0.578) improved the diagnostic efficiencies of individual preoperative examinations. Based on the high sensitivity of IFS, parallel strategies improved the sensitivities of preoperative examinations to 89.66% (MRI), 64.18% (D&C), 62.69% (Hys), respectively, and these differences were statistically significant (*p* = 0.000).

**Conclusion:**

IFS presented reasonable agreement rates predicting postoperative PS results, including deep myometrial invasion and high‐grade. IFS helps identify high‐intermediate risk patients in preoperative biopsy and MRI and guides intraoperative lymphadenectomy decisions in EA.

## Graphical abstract

In this multi‐center retrospective study, the authors proposed that it is not the time to abandon intraoperative frozen section in endometrioid adenocarcinoma. Intraoperative frozen section presented reasonable agreement rates predicting postoperative pathology, including deep myometrial invasion and high‐grade. Intraoperative frozen section helps identify high‐intermediate risk patients in preoperative biopsy and MRI and guides intraoperative lymphadenectomy decisions in endometrioid adenocarcinoma.

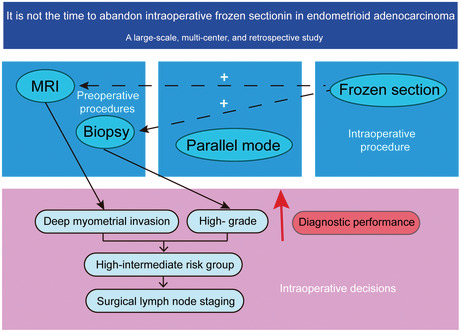

## 1 INTRODUCTION

Endometrioid adenocarcinoma (EA) is a common malignant tumor of the female reproductive tract in developed countries.^1^ Although most EA women have favorable prognoses, patients with deep myometrial invasion (≥ 50% of myometrial thickness), high‐grade (grade 3), substantial lymph vascular space invasion (LVSI), and cervical stromal involvement have higher lymphatic involvement risks.^2^ Consequently, pre/intraoperative methods to evaluate high‐risk factors are urgent for surgical decisions. Some gynecological centers use intraoperative frozen section (IFS) for better detection. However, current studies have presented conflicting insights on IFS accuracy.^3^, ^4^, ^5^, ^6^, ^7^, ^8^, ^9^, ^10^ The 2021 European Society of Gynecological Oncology (ESGO) guidelines are reluctant to recommend IFS due to repetition inability, interference with adequate pathological processing.^2^ The failure to reach a consensus on whether IFS should not be used has caused a long dispute in Chinese academic circles. When there is a discrepancy between different examinations, surgeons can have difficulty determining priorities and perform composite diagnoses. Therefore, IFS can be used as part of composite diagnoses to instruct clinical procedures and pathological diagnosis for EA. Besides, most studies have not fully described the factors affecting IFS diagnostic accuracy. Therefore, we assessed IFS accuracy in predicting postoperative paraffin section (PS) results, including deep myometrial invasion and high‐grade, compared to other pre/intraoperative methods. We determined to what degree addition of IFS results improves identification of high‐risk factors by routine preoperative examinations and clarified the clinical factors of IFS misdiagnosis.

## 2 MATERIALS AND METHODS

### 2.1 Study population

**FIGURE 1:**
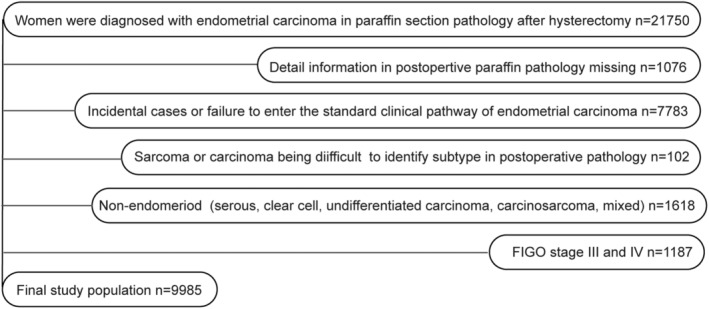
Flowchart showing the inclusion and exclusion of study participants. The endometrial carcinoma database had 21,750 cases accurately diagnosed in postoperative pathology; 1,076 cases without postoperative pathology details were excluded, including localized endometrial carcinoma lesions only confined to the endometrium layer. Lesions were removed during preoperative biopsies, and no cancer lesions were found in postoperative paraffin pathological sections. We excluded 7,783 incidental cases or cases out of the standard clinical pathway of endometrial carcinoma. Some cases were accidentally found with endometrial carcinoma during uterus intraoperative gross evaluation (IGE), IFS, or postoperative pathology, which did not undergo the standardized clinical pathway of endometrial carcinoma and standardized pre/intraoperatively high‐risk factors assessments. Included patients were preoperatively diagnosed based on a previous biopsy or were observed with definite intrauterine occupations in preoperative imaging or ultrasound. Admission diagnoses comprised endometrial carcinoma, occupation disease in the uterine cavity or atypical hyperplasia (possible endometrial carcinoma). We excluded 102 sarcomas or cases difficult to diagnose. In the remaining 12,789 participants, we excluded 1,618 patients with non‐endometrioid endometrial carcinoma (serous, clear cell, undifferentiated, mixed carcinoma, and carcinosarcoma). After excluding 1,187 cases in clinical‐stage III/IV, 9,985 EA women who underwent pre/intraoperatively high‐risk factors assessment were retrieved for subsequent analysis.

Consecutive 21,750 patients who underwent hysterectomy and diagnosed with endometrial carcinoma between January 2000 and December 2019 at 30 hospitals in China were recruited. 1,076 patients were excluded due to incomplete postoperative assessment data. 7,783 incidental cases were excluded due to non‐standard clinical pathway of endometrial carcinoma. Patients who had sarcomas or unknown pathology (*n* = 102), or coexisting non‐endometrioid components (*n* = 1618) were also excluded. After excluding 1,187 cases in advanced stage, 9,985 endometrioid adenocarcinoma women who underwent pre/intraoperatively high‐risk factors assessments were retrieved for subsequent analysis (Figure 1). Institutional Review Boards approved this study in all centers.

### 2.2 Definitions and study design

After admission, the 9,985 patients were examined and treated according to the clinical pathway of endometrial carcinoma. We collected clinical data, including personal history, previous history, menstruation, marriage, and childbearing history, operation history, pre/intraoperative examination, and final postoperative pathology. We re‐checked data according to computed tomography (CT), magnetic resonance imaging (MRI), intraoperative gross examination (IGE), IFS, diagnostic curettage (D&C), hysteroscopy (Hys), and postoperative PS standardized protocols to exclude diagnostic bias from different institutions. Diagnostic criteria were detailed in Supplementary Materials S1. No myometrial invasion comprises microscopic lesions confined to the endometrium (no myometrial invasion). Superficial myometrial invasion represents a depth of lesion involvement <1/2 of the myometrium microscopically (<50%).^11^ Deep myometrial invasion comprises a depth of lesion involvement ≥1/2 of the myometrium (≥ 50%). High‐grade represents grade 3 based on accurately identifying EA.^2^

We compared the diagnostic ability in detecting deep myometrial invasion and high‐grade between IFS and other pre/intraoperative procedures (MRI, CT, IGE, D&C, and Hys), considering PS pathology after hysterectomy as the gold standard. Moreover, we explored possibilities of adopting IFS as auxiliary tools in preoperative examinations. Lastly, we clarified factors related to IFS misdiagnosis. The 9,985 women were further screened into different groups.

### 2.3 Statistical analysis

SPSS 27.0 (IBM SPSS Statistics for Windows Armonk) was used for statistical analyses. Diagnostic efficacy was calculated using accuracy, sensitivity, specificity, positive predictive value (PPV), and negative predictive value (NPV), with 95% confidence intervals (CIs). We used the Mc‐Nemar test to compare sensitivity and specificity differences. We tested the agreement between two methods using the Kappa test and Cronbach's α‐inter rate correlation. We used a bivariate logistic regression model to evaluate risk factors for IFS diagnostic errors. The −2 log‐likelihood ratio (LR) was used for the model's overall significance. Hosmer–Lemeshow goodness‐of‐fit *χ* ^2^ test assessed model fit. Results are expressed as odds ratios (ORs) with 95% CIs and *p*‐values. A *p* < 0.05 was considered significant. Statistical indicators are detailed in Table S1.

## 3 RESULTS

### 3.1 Final cohort

The baseline characteristics of the 9,985 EA women are presented in Table 1. The median age at diagnosis was 54.08 ± 9.28 years (range: 17–92 years); 8,844 (88.57%) cases had preoperative D&C or Hys. More than half of patients were postmenopausal (57.31%). The most common disease‐related triads in EA was hypertension (42.52%), followed by diabetes (12.53%), and obesity (12.19%). Only 12.07% of patients had myoma or adenomyosis; 5,398 (54.06%) cases had a laparoscopy and 4,576 (45.83%) a laparotomy. The prevalence of deep myometrial invasion was 16.46%, based on the final histopathology. Most women (86.44%) had low‐risk grades, and only 10.44% EA showed atypical hyperplasia elements in postoperative pathology.

**TABLE 1:** Clinical and pathological characteristics of the study population.

Study population		*N* = 9985 (%) for cases
Age at diagnosis (year), median ± standard error		54.08 ± 9.28
Preoperative biopsy	Yes	8,844 (88.57%)
	No	1,141 (11.43%)
Menopause	Pre	3,900 (39.06%)
	Post	5,722 (57.31%)
	Unknown	363 (3.63%)
Diabetes	No	8,734 (87.47%)
	Yes	1,251 (12.53%)
Hypertension	No	5,739 (57.48%)
	Yes	4,246 (42.52%)
Obesity	No	8,768 (87.81%)
	Yes	1,217 (12.19%)
Uterine diseases	No	8,780 (87.93%)
	Myoma	913 (9.14%)
	Adenomyosis	157 (1.58%)
	Both	135 (1.35%)
Surgery	Laparotomy	4,576 (45.83%)
	Laparoscope	5,398 (54.06%)
	Others	11 (0.11%)
Myometrial invasion	No/<50%	8,341 (83.54%)
	≥50%	1,644 (16.46%)
Atypical hyperplasia	No	8,943 (89.56%)
	Yes	1,042 (10.44%)
Histological grade	Low grade	8,631 (86.44%)
	High grade	1,354 (13.56%)

### 3.2 Deep myometrial invasion assessment

**FIGURE 2:**
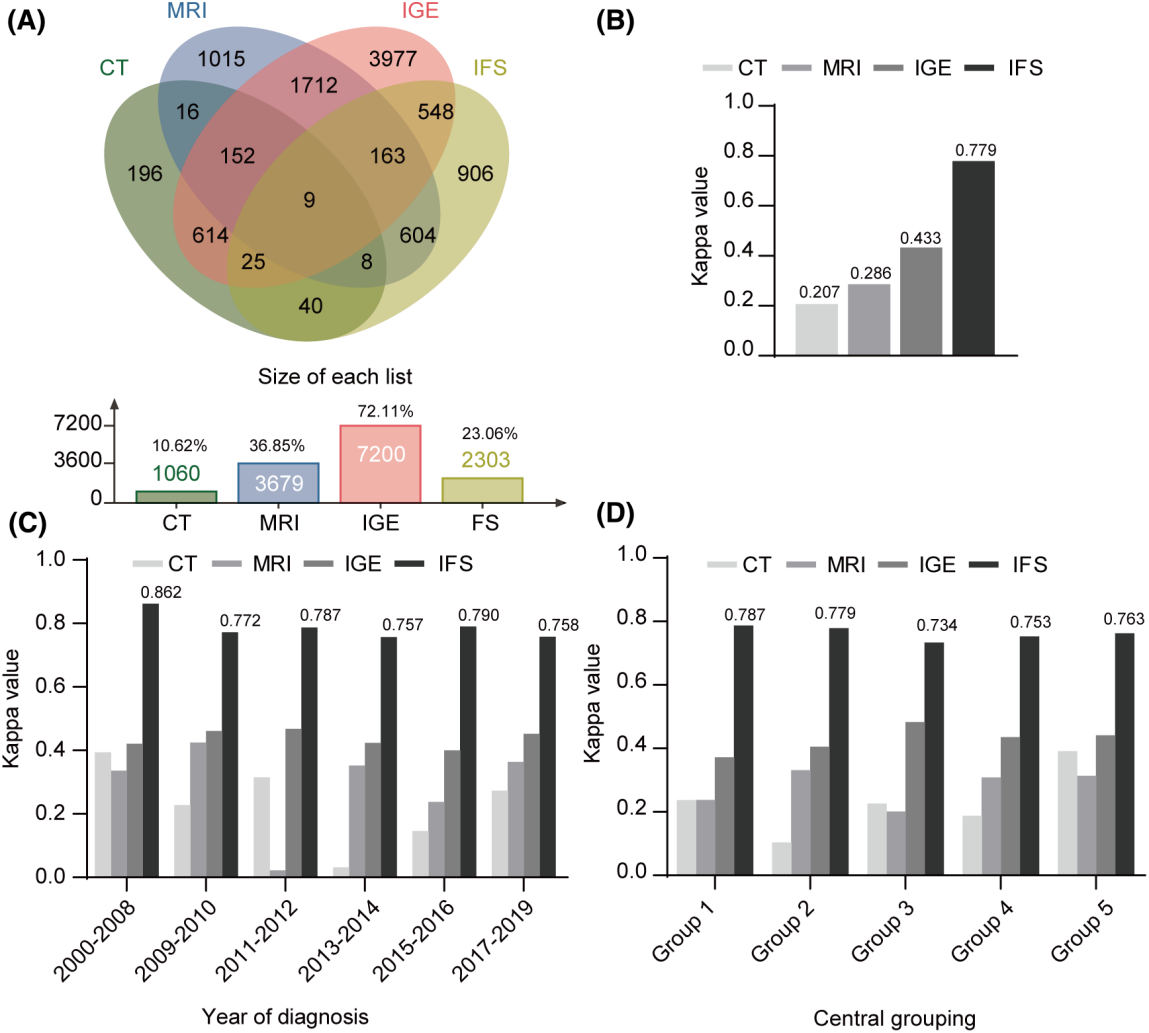
Graphic representation of the utility differences in detecting deep myometrial invasion between CT, MRI, IGE, and IFS. (A) Wayne chart representation of different methods (CT, MRI, IGE, and IFS). (B) Histogram representation of the differences in diagnosis efficacy (Kappa values) between CT, MRI, IGE, and IFS. (C) Stratified consistency analyses based on the diagnosis year. (D) Consistency analyses grouped by medical centers.

We included 2,303 (23.06%) women with myometrial invasion assessments by IFS, 7,200 (72.11%) by IGE, 3,679 (36.85%) by MRI, and 1,060 (10.62%) by CT (Figure 2A). The highest consistency was observed between IFS and the final pathology report (Kappa: 0.779), demonstrating the high IFS repeatability (Figure 2B), followed by IGE (Kappa: 0.433), MRI (Kappa: 0.286), and CT (Kappa: 0.207). Kappa values (CT, MRI, IGE, and IFS) were divided by the diagnosis year (Figure 2C). No variations were detected in IFS diagnostic efficiency between 2000 and 2019. IFS maintained favorable diagnoses compared to CT, MRI, and IGE. Stratified consistency analyses based on the diagnosis year are shown in Tables S2 and S3. We grouped data by medical centers (Table S4) and performed consistency checks (Figure 2D and Table S5). Although different centers have unique inspection standards, no evident variations were observed between centers for IFS diagnostic efficiency. Many factors influence the selection in pre‐ and intra‐operative work‐up and must be performed in the same group to compare the diagnostic effectiveness of various methods to reduce sample selection bias (Table 2). Hence, we retrieved 172 women who all received MRI, IGE, and IFS to evaluate deep myometrial invasion. The consistency between IFS and final pathology was higher (Cronbach's *α*: 0.910; Kappa: 0.834) than IGE (Cronbach's *α*: 0.782; Kappa: 0.643) and MRI (Cronbach's *α*: 0.566; Kappa: 0.395). High IFS sensitivity (86.21%) supports avoiding underdiagnosis compared to IGE (68.97%) and MRI (51.72%). IFS (97.20%), IGE (94.41%), and MRI (88.81%) specificity were around 90%.

**TABLE 2:** Myometrial invasion correlation between MRI, IGE, IFS, parallel mode (MRI + IFS), parallel mode (IGE + IFS), and final surgical specimen.

MI	Postoperative PS			Cronbach's α	Kappa	Accuracy% (95% CI)	Sensitivity % (95% CI)	Specificity % (95% CI)	PPV % (95% CI)	NPV % (95% CI)
	No/<50%	≥50%	Total							
MRI										
No/<50%	127	14	141	0.566	0.395	82.56 (75.88–87.75)	51.72 (32.9–70.11)	88.81 (82.19–93.27)	48.39 (30.56–66.60)	90.07 (83.60–94.26)
≥50%	16	15	31							
Total	143	29	172							
MRI + IFS in parallel										
No/<50%	124	3	127	0.782	0.626	87.21 (81.06–91.64)	89.66 (71.51–97.29)	86.71 (79.78–91.60)	57.78 (42.24–72.01)	97.64 (92.73–99.39)
≥50%	19	26	45							
Total	143	29	172							
IGE										
No/<50%	135	9	144	0.782	0.643	90.12 (84.41–93.97)	68.97 (49.05–84.03)	94.41 (88.91–97.38)	71.43 (51.13–86.05)	93.75 (88.12–96.92)
≥50%	8	20	28							
Total	143	29	172							
IGE + IFS in parallel										
No/<50%	135	4	139	0.867	0.764	93.02 (87.85–96.18)	86.21 (67.43–95.49)	94.41 (88.91–97.38)	75.76 (57.37–88.26)	97.12 (92.34–99.07)
≥50%	8	25	33							
Total	143	29	172							
IFS										
No/<50%	139	4	143	0.910	0.834	95.35 (90.72–97.83)	86.21 (67.43–95.49)	97.20 (92.55–99.10)	86.21 (67.43–95.49)	97.20 (92.55–99.10)
≥50%	4	25	29							
Total	143	29	172							

Abbreviations: CT, computerized tomography; IFS, intraoperative frozen section; IGE, intraoperative gross examination; MI, myometrial invasion; MRI, magnetic resonance imaging; PS, paraffin section.

Since IFS sensitivity was higher than MRI, we performed parallel diagnoses between IFS and MRI, which can further improve sensitivity but decrease diagnostic specificity (Table 2). MRI and IFS combined (89.66%) significantly increased MRI sensitivity (51.72%, *p* = 0.000). The combined pattern reduced specificity (86.71%) compared to MRI alone (88.81%) without significance (*p =* 0.125). However, sensitivity and specificity assessments are only part of overall evaluations. Parallel strategies (MRI‐IFS: Kappa: 0.626) did improve the consistency of MRI alone (Kappa: 0.395).

### 3.3 High‐grade evaluation

**FIGURE 3:**
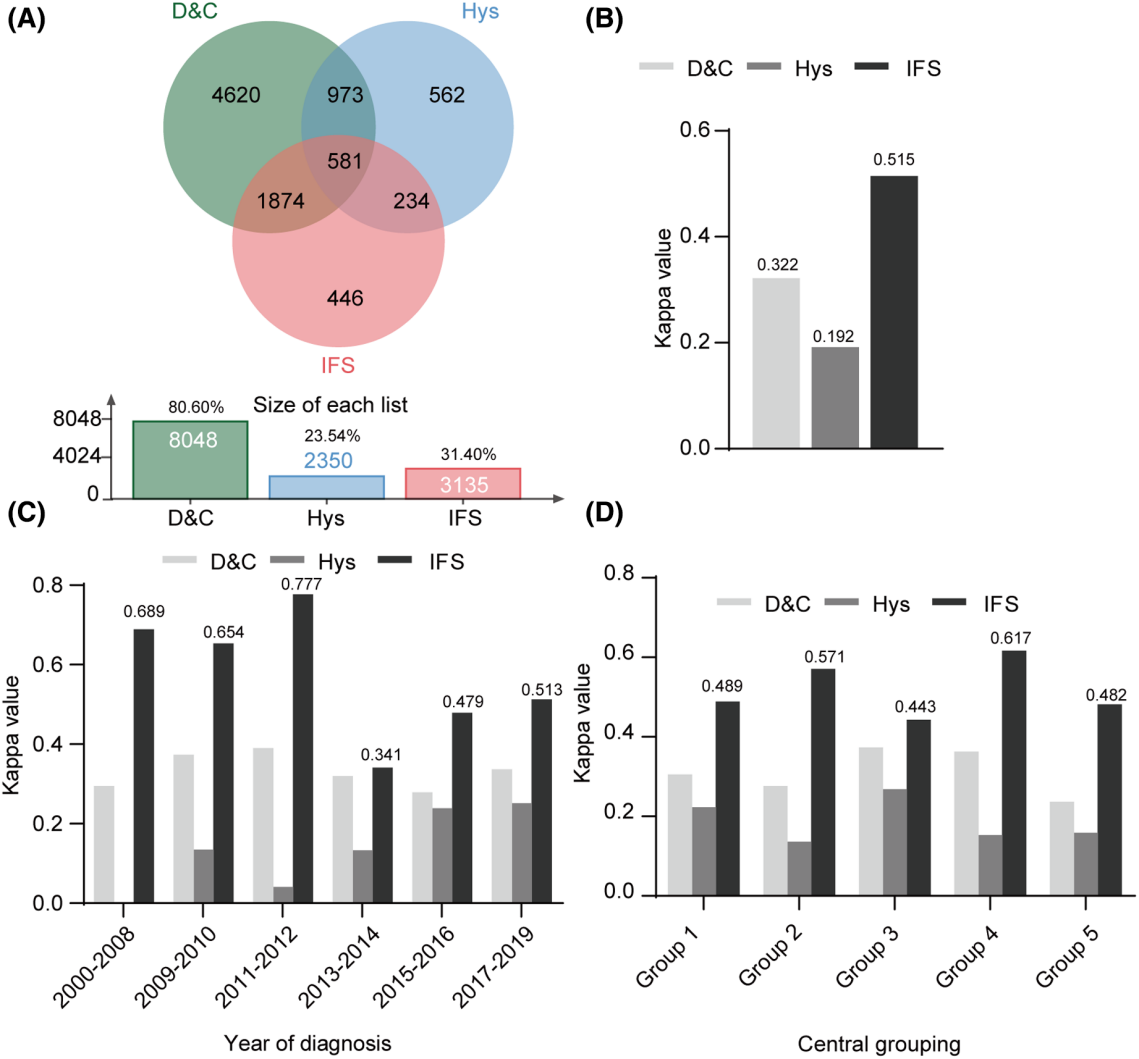
Graphic representation of the utility differences in detecting endometroid adenocarcinoma with grade 3 between D&C, Hys, and IFS. (A) Wayne chart representation of different methods (D&C, Hys and IFS). (B) Histogram representation of the differences in diagnosis efficacy (Kappa values) between D&C, Hys, and IFS. (C) Stratified consistency analyses based on the diagnosis year. (D) Consistency analyses grouped by medical centers.

We included 8,048 (80.60%) women with high‐grade assessments by D&C, 2,350 (23.54%) by Hys, and 3,135 (31.40%) by IFS (Figure 3A). IFS presented the highest consistency (Kappa: 0.515), followed by D&C (Kappa: 0.322) and Hys (Kappa: 0.192) (Figure 3B). IFS maintained effective functions for high‐grade diagnoses compared to D&C and Hys in different years and centers (Figures 3C, D). Stratified consistency analyses based on the diagnosis year and centers are presented in Tables S6 and S7. To evaluate high‐grade, 581 women received D&C, Hys, and IFS (Table 3). IFS and final pathology presented a higher consistency (Cronbach's α: 0.739; Kappa: 0.585) than D&C (Kappa: 0.226) and Hys (Kappa: 0.180). The IFS (96.50%), D&C (98.83%), and Hys (98.64%) specificity were all above 95%. Only IFS sensitivity reached 50% (58.21%) [D&C (16.42%), Hys (13.43%)]. To improve preoperative biopsy sensitivity, we performed a parallel diagnosis (D&C‐IFS: 64.18%; Hys‐IFS: 62.69%) and demonstrated the significance compared with biopsy alone (*p* = 0.000). This combination (D&C‐IFS Kappa: 0.595; Hys‐IFS Kappa: 0.578) did significantly improve the consistency of biopsy alone, both with moderate consistency.

**TABLE 3:** Tumor grade correlation between D&C, Hys, IFS, parallel mode (D&C + IFS), parallel mode (Hys + IFS), and final surgical specimen.

High‐grade EA	Postoperative PS			Cronbach's α	Kappa	Accuracy% (95% CI)	Sensitivity % (95% CI)	Specificity % (95% CI)	PPV % (95% CI)	NPV % (95% CI)
	No	Yes	Total							
D&C										
No	508	56	564	0.385	0.226	89.33 (86.46–91.66)	16.42 (8.87–27.91)	98.83 (97.34–99.52)	64.71 (38.62–84.74)	90.07 (87.23–92.35)
Yes	6	11	17							
Total	514	67	581							
D&C + IFS in parallel										
No	490	24	514	0.746	0.595	91.74 (89.12–93.79)	64.18 (51.47–75.26)	95.33 (93.03–96.92)	64.18 (51.47–75.26)	95.33 (93.03–96.92)
Yes	24	43	67							
Total	514	67	581							
Hys										
No	507	58	565	0.321	0.180	88.81 (85.89–91.20)	13.43 (6.70–24.47)	98.64 (97.09–99.40)	56.25 (30.55–79.25)	89.73 (86.86–92.05)
Yes	7	9	16							
Total	514	67	581							
Hys + IFS in parallel										
No	489	25	514	0.733	0.578	91.39 (88.73–93.48)	62.69 (49.97–73.95)	95.14 (92.81–96.77)	62.69 (49.97–73.95)	95.14 (92.81–96.77)
Yes	25	42	67							
Total	514	67	581							
IFS										
No	496	28	524	0.739	0.585	92.08 (89.50–94.08)	58.21 (45.54–69.94)	96.50 (94.42–97.85)	68.42 (54.62–79.73)	94.66 (92.28–96.36)
Yes	18	39	57							
Total	514	67	581							

Abbreviations: D&C, diagnostic curettage; IFS, intraoperative frozen section; High‐grade EA, endometrioid adenocarcinoma with grade 3; Hys, hysteroscope; PS, paraffin section.

### 3.4 Independent factors associated with IFS mis‐diagnosis

Women with IFS accurate, under, and over‐diagnoses of deep myometrial invasion were compared regarding menopausal status, atypical hyperplasia, intrauterine diseases (myoma or adenomyosis), cesarean section, abortion history, and histologic grade (low and high) (Table 4). The multivariate analysis showed that post‐menopause (OR 1.644; 95% CI: 1.034–2.614; *p* = 0.036) indicated risks for IFS under‐diagnosis of deep myometrial invasion (Table 5). No significant association was detected between menopause, atypical hyperplasia, cesarean section, and abortion history with high‐grade IFS misdiagnosis (Table 6). In the multivariate analysis (Table 7), deep myometrial invasion probability was significantly higher for under‐diagnoses than accurate diagnoses (OR 1.502; 95% CI: 1.034–2.183; *p* = 0.033), as well as myoma or adenomyosis probability for over‐diagnoses than accurate diagnoses (OR 1.935; 95% CI: 1.106–3.388; *p* = 0.021).

**TABLE 4:** Univariate logistic regression analysis of “IFS under‐diagnoses or not” and “IFS over‐diagnoses or not” in detecting deep myometrial invasion.

IFS detecting deep MI	Accurate‐diagnoses	Under‐diagnoses	Under‐diagnoses or not		Over‐diagnoses	Over‐diagnoses or not	
			OR 95% CI	*p* Value		OR 95% CI	*p* Value
Menopause							
Pre	875 (40.05)	26 (28.89)	1	0.036	7 (30.43)	1	0.353
Post	1,310 (59.95)	64 (71.11)	1.644 (1.034–2.614)		16 (69.57)	1.527 (0.626–3.726)	
Atypical hyperplasia							
No	1,905 (87.03)	77 (85.56)	1	0.685	20 (83.33)	1	0.594
Yes	284 (12.97)	13 (14.44)	1.132 (0.621–2.065)		4 (16.67)	1.342 (0.455–3.953)	
Uterine diseases (myoma or adenomyosis)							
No	1,971 (90.04)	85 (94.44)	1	0.175	21 (87.50)	1	0.680
Yes	218 (9.96)	5 (5.56)	0.532 (0.213–1.325)		3 (12.50)	1.292 (0.382–4.365)	
Caesarean section							
No	1,962 (89.79)	85 (94.44)	1	0.157	19 (82.61)	1	0.266
Yes	223 (10.21)	5 (5.56)	0.518 (0.208–1.289)		4 (17.39)	1.852 (0.625–5.493)	
Abortion history							
No	892 (40.82)	31 (34.44)	1	0.228	12 (52.17)	1	0.275
Yes	1,293 (59.18)	59 (65.56)	1.313 (0.843–2.045)		11 (47.83)	0.632 (0.278–1.440)	
Histological grade							
Low	1,931 (88.21)	76 (84.44)	1	0.282	21 (87.50)	1	0.914
High	258 (11.79)	14 (15.56)	1.379 (0.768–2.474)		3 (12.50)	1.069 (0.317–3.610)	

Abbreviations: IFS, intraoperative frozen section; High grade, grade 3; Low grade, grade 1/2; MI, myometrial invasion; OR, dominance ratio.

**TABLE 5:** Multivariate logistic regression analysis of “IFS under‐diagnoses or not” in detecting deep myometrial invasion.

Characteristics	B	SE	Wald	*p* Value	OR (95% CI)
	IFS under‐diagnoses of deep MI				
Menopause pre versus post	0.497	0.237	4.415	0.036	1.644 (1.034–2.614)

Abbreviations: B, partial regression coefficient B; IFS, intraoperative frozen section; MI, myometrial invasion; OR, dominance ratio; SE, standard error; Wald, Wald statistic.

**TABLE 6:** Univariate logistic regression analysis of “IFS under‐diagnoses or not” and “IFS over‐diagnoses or not” in detecting high‐grade.

IFS detecting high‐grade EA	Accurate‐diagnoses	Under‐diagnoses	Under‐diagnoses or not		Over‐diagnoses	Over‐diagnoses or not	
			OR 95% CI	*p* Value		OR 95% CI	*p* Value
Menopause							
Pre	1,215 (42.77)	75 (38.07)	1	0.198	43 (53.75)	1	0.052
Post	1,626 (57.23)	122 (61.93)	1.215 (0.903–1.636)		37 (46.25)	0.643 (0.412–1.004)	
Atypical hyperplasia							
No	2,368 (82.94)	170 (85.43)	1	0.366	68 (83.95)	1	0.812
Yes	487 (17.06)	29 (14.57)	0.829 (0.553–1.244)		13 (16.05)	0.930 (0.510–1.696)	
Uterine diseases (myoma or adenomyosis)							
No	2,530 (88.62)	180 (90.45)	1	0.429	65 (80.25)	1	0.023
Yes	325 (11.38)	19 (9.55)	0.822 (0.505–1.337)		16 (19.75)	1.916 (1.096–3.351)	
Caesarean section							
No	2,542 (89.48)	173 (87.82)	1	0.466	70 (87.50)	1	0.572
Yes	299 (10.52)	24 (12.18)	1.179 (0.757–1.838)		10 (12.50)	1.215 (0.619–2.381)	
Abortion history							
No	1,185 (41.71)	87 (44.16)	1	0.500	34 (42.50)	1	0.888
Yes	1,656 (58.29)	110 (55.84)	0.905 (0.676–1.210)		46 (57.50)	0.968 (0.618–1.518)	
Deep MI							
No	2,476 (86.73)	162 (81.41)	1	0.036	71 (87.65)	1	0.808
Yes	379 (13.27)	37 (18.59)	1.492 (1.027–2.167)		10 (12.35)	0.920 (0.470–1.800)	

Abbreviations: IFS, intraoperative frozen section; High‐grade EA, endometrioid adenocarcinoma with grade 3; MI, myometrial invasion; OR, dominance ratio.

**TABLE 7:** Multivariate logistic regression analysis of “IFS under‐diagnoses or not” and “IFS over‐diagnoses or not” in detecting high‐grade.

Characteristics	B	SE	Wald	*p* Value	OR (95% CI)
	IFS under‐diagnoses of high‐grade EA				
Deep MI no versus yes	0.407	0.191	4.559	0.033	1.502 (1.034–2.183)
	IFS over‐diagnoses of high‐grade EA				
Uterine diseases no versus yes	0.660	0.286	5.343	0.021	1.935 (1.106–3.388)

Abbreviations: B, partial regression coefficient B; IFS, intraoperative frozen section; High‐grade EA, endometrioid adenocarcinoma with grade 3; MI, myometrial invasion; OR, dominance ratio; SE, standard error; Wald, Wald statistic.

## 4 DISCUSSION

According to 2021 ESGO guidelines, stage I with substantial LVSI, stage II, and stage IB (deep myometrial invasion) high‐grade EA, regardless of LVSI status, are classified into a high‐intermediate risk group and need surgical lymph node staging.^2^ Sentinel lymph node (SLN) biopsy is acceptable for systematic lymphadenectomy when lesions are confined to the uterus in high/high‐intermediate women.^2^, ^11^ About 50% of surgeons adopt SLN biopsies, widely used in 69 countries (mostly from Europe and the USA).^12^ However, some medical centers cannot perform SLNs. Moreover, accurately mapping SLNs still has some challenges. Pathologic ultrastaging based on H&E staining allows accurate identification of SLN metastases but delays the final diagnosis due to complex tissue processing and staining.^13^, ^14^ Thus, tools for selecting high‐risk factors in centers without SLN procedures are crucial. The 2021 ESGO guidelines recommend that histopathologic tumor type and grade information refer to the biopsy and myometrial invasion assessment refer to the pelvic MRI or transvaginal sonography (TVS). However, IFS is not encouraged for myometrial invasion assessment because of poor reproducibility.^2^ However, socioeconomic statuses, medical levels, and clinical strategies vary between Chinese and Western regions. Therefore, investigating the most practical method for high‐risk assessments is essential in the Chinese population. Our results suggested that the advantage of IFS is mainly reflected by significantly higher sensitivity and reproducibility for detecting deep myometrial invasion and high‐grade. The specificity difference was not evident, with both reaching about 90%. IFS can be used as an additional method to increase the diagnostic effectiveness of preoperative examinations in parallel mode. It is not the time to completely abandon IFS in endometrioid adenocarcinoma. We also demonstrated that some non‐native guidelines hardly apply to local women. For decision‐making, domestic experts should use retrospective studies with local women at proper proportions.

IGE is relatively fast and accurate for myometrial invasion and helpful for determining high‐risks,^4^, ^15^, ^16^ consistent with our results that IGE consistency was substantial but remained inferior to IFS. Lesion's skip metastasis is difficult to identify on IGE,^17^ resulting in some missed diagnoses. IFS is more accurate than IGE because physicians must take complete samples from the entire endometrial cavity and perform more detailed sectional examinations.^18^ MRI is also preoperatively used to evaluate myometrial invasion depth. Many studies have presented inconsistent MRI sensitivity (33%–88%) and specificity (74%–100%) for myometrial invasion.^19^, ^20^ Previously published criteria recommend myometrial invasion assessment via T2WI and dynamic images.^21^, ^22^ A meta‐analysis showed that T2WI sequences have sensitivity of 72%, specificity of 82%, PPV of 58%, and NPV of 90%.^23^ Herein, MRI had sensitivity of 51.71% and specificity of 88.81%, low compared to foreign research. Differences in medical imaging devices, radiological technology, and reading ability training in clinical practice might result in “MRI defects in China”. Generally, IFS performs better than preoperative MRI for myometrial invasion in endometrial cancer. However, MRI with diffusion‐weighted imaging has similar accuracy to IFS.^24^ Due to limited retrospective data, we could not select diffusion‐weighted or dynamic imaging and compare with IFS. Although with good accuracy in some medical centers, MRI remains expensive and not always available, especially in developing countries. Some studies echoed significant CT flaws, consistent with our results. Few studies have recommended CT for myometrial invasion, widely used for extrauterine lesions and lymph node enlargement.^25^, ^26^

Clinical IFS application for EA remains controversial, presenting sensitivity of 74%–93%, specificity of 95%–97%, and accuracy of 89%–94%.^19^, ^24^, ^27^, ^28^ In two prospective blinded accuracy evaluations, IFS presented a high under‐staging risk, resulting in inadequate treatment.^29^, ^30^ Frumovitz et al. reported that IFS for myometrial invasion depth was not well correlated to final pathology.^8^ We speculate that the discordance could be attributable to the fact that the selected specimen is not always representative for analysis of the deepest MI. We believe one reason for IFS poor reproducibility (mentioned in 2021 ESGO guidelines) related to bias in selection of specimens for frozen section. Here, the controversy over IFS is mainly related to the different feasibilities based on pathologists' skills. We found that IFS had higher sensitivity (86.21%), specificity (97.20%), and agreement rate (Kappa: 0.834) for deep myometrial invasion than overall levels, which might be derived from the fact that some cases may refer to preoperative imaging results and perform targeted frozen section sampling. However, the dominance and significance of IFS have not noticeably reduced in the total IFS crowd (with or without preopreative imaging) (N: 2303; Kappa: 0.779), compared with women who concurrently received MRI, IGE, and IFS (N: 172; Kappa: 0.834). Besides, the endometrium penetrates the basal layer without clear boundaries in standard anatomical structure. Its more likely to be underdiagnosed in MRI and IGE when the EA lesion is small or at the junction. For IFS, tissues can be widely sampled and cut into thin slices (few microns).^18^ Thus, “IFS has repeatability advantages in China”, different from previous studies. Herein, IFS underdiagnoses were associated with a higher post‐menopause proportion. Thus, we hypothesized that potential postmenopausal endometrial and muscular atrophy artifacts contribute to IFS underdiagnoses of deep myometrial invasion. We explored the correlation between detailed uterine anatomy measurements and underdiagnoses, but without success.

Grade information can be obtained via preoperative biopsy.^2^ There is no complete agreement between preoperative grade and final histopathology, affected by parameters such as sampling method and tumor diameter.^31^ A previous review reported a discrepancy of 35.9%–7.0% and 20.0%–16.2% on the final diagnosis for the D&C and Hys, respectively.^32^ Regarding concordance, similar to the biopsy, there is some discordance between IFS and postoperative pathology.^33^ Tumor grade evaluation by IFS has presented sensitivity of 40%–98%, specificity of 53%–98%, accuracy of 83%–89%, and correlation of 58%–88%.^19^, ^24^, ^27^ Herein, the overall IFS consistency was moderate for postoperative pathology results (Kappa: 0.585) compared to D&C (0.226) and Hys (0.180). We found very low sensitivity in preoperative biopsies (blind or hysteroscopically guided), resulting in many underdiagnoses, possibly from inadequate sampling, especially when high‐grade lesions tend to invade the deep myometrium. The relatively higher sensitivity (58.21%) and consistency of IFS might be partially derived from the fact that cases may refer to preoperative biopsy results in the evaluation of IFS. Nevertheless, we found that the agreements of IFS in the total crowd with or without biopsy (N: 3135; Kappa: 0.515) and in cases who concurrently received D&C, Hys, and IFS (N: 581; Kappa: 0.585) both reached moderate levels. Neither preoperative biopsy nor IFS provided high consistency with postoperative pathology, which might be related to our high‐grade definition (grade 3) based on accurate EA identification. Due to the relatively high IFS misdiagnosis rate, especially underdiagnosis probability (41.79%), we compared the characteristics of women with different diagnoses (IFS accuracy vs. under/overdiagnosis). High‐grade underdiagnoses were associated with a higher deep myometrial invasion proportion, and high‐grade overdiagnoses with a higher myoma or adenomyosis proportion. Although no studies have evaluated the factors associated with IFS high‐grade misdiagnosis, Santoro et al. found that IFS usually underestimates tumor grade rather than overestimates.^34^ Considering insufficient sampling or artifacts that might disrupt nuclear atypia, it is essential to eliminate these conditions during IFS.^35^

Although our results illustrated that IFS is reasonable for detecting deep myometrial invasion and high‐grade, many researches have suggested that IFS is not encouraged because of interference with adequate pathological processing such as molecular classification.^2^ The 2021 ESGO guidelines recommend that the molecular classification, including three immunohistochemical markers (p53, MSH6, and PMS2) and one molecular test (mutation analysis of the exonuclease domain of POLE) should be encouraged in all endometrial carcinomas, although data regarding integrated molecular and histological prognostic factors remain scarce.^2^, ^36^ Indeed, some of the proposed biomarkers require high‐quality preanalytical treatment of surgical specimens, such as appropriate fixation conditions. So, there is a trade‐off between the diagnostic priority IFS and the risk of interfering with pathological processing. Due to the limited application of the Proactive Molecular Risk Classifier for Endometrial Cancer (ProMisE) in China, evaluating the risk factors with unknown molecular classification for endometrial carcinoma is still an important step in the diagnosis and treatment of endometrial carcinoma. Assessing deep myometrial invasion and high‐grade during surgery to guide the excision extent is a priority for some patients, especially with skeptical preoperative results, and inexpensive and readily available IFS might be a better option.

Our study has five main limitations. First, we did not include myometrial invasion assessments by TVS. In the 30 included centers, partial clinicians tended to choose non‐invasive MRI rather than invasive TVS before operation, especially when patients still had vaginal bleeding, leading to details missing in most TVS reports. Second, we did not consider other risk factors important for lymph node metastasis (e.g., LVSI or cervical involvement). Hence, our conclusions are not definitive. It has been demonstrated extensively that MRI techniques are highly specific in the assessment of cervical stromal involvement.^2^ The IFS diagnostic performance is still controversial. Accuracy of IFS in determining cervical involvement was reported as 86.7%–100% in literature.^33^ Karabagli et al. reported 100% correlation between IFS and PS.^37^ However, Gülşen et al. reported that cervical involvement was detected with 50% sensitivity and 97.6% specificity.^33^ The low sensitivity can be explained by the limited cervical examination at IFS. The lack of cervical involvement in some IFS reports resulted in our failure to evaluate this parameter. Moreover, IFS can lead to artifactual displacement of tumor cells into vascular spaces, resulting in incorrect assessment of LVSI.^2^ Therefore, LVSI was not evaluated in this study. Third, the retrospective and multi‐center design with a large time span were limiting, especially retrospective data from institutions with different examination protocols. We worked with several centers to develop a unique protocol to re‐check and review data. We also stratified diagnostic effectiveness by year and center, and IFS efficiency remained favorable compared to other methods, demonstrating effective data validation. Fourth, the pathologists who made the final diagnosis were aware of IFS results in most cases, which might lead to bias for IFS. Finally, one limitation of this report is the lack of randomization for any observational design that can mislead for selection bias, although a double‐blind review of the results was performed. Therefore, high sensitivity of IFS regarding these parameters in our cases may be explained by the fact that IFS is not routinely performed for EA, only for reconfirming the suspicious results in preoperative examinations.

## 5 CONCLUSION

In summary, IFS can identify deep myometrial invasion and high‐grade with high sensitivity, specificity, and repeatability. This advantage can be used to improve the efficiencies of MRI and preoperative biopsy for identification of high‐risk factors. IFS remains acceptable to guide intraoperative decisions for lymphadenectomy, especially when the preoperative examination has ambiguous results. Women with post‐menopause status or deep myometrial invasion need to guard against the underdiagnosis for deep myometrial invasion and advanced grade. Besides, equipment renewal and additional education on imaging should be provided, as well as strengthen TVS popularization for EA high‐risk factors.

## AUTHOR CONTRIBUTIONS

**Xiaohang Yang:** Formal analysis (equal); investigation (equal); methodology (equal); software (lead); validation (lead); visualization (lead); writing – original draft (lead); writing – review and editing (lead). **Jingjing Yin:** Formal analysis (equal). **Yu Fu:** Formal analysis (equal). **Yuanming Shen:** Resources (equal); supervision (equal). **Chuyao Zhang:** Resources (equal); supervision (equal). **Shuzhong Yao:** Resources (equal); supervision (equal). **Congjian Xu:** Resources (equal); supervision (equal). **Min Xia:** Resources (equal); supervision (equal). **Ge Lou:** Resources (equal); supervision (equal). **Jihong Liu:** Resources (equal); supervision (equal). **Bei Lin:** Resources (equal); supervision (equal). **Jianliu Wang:** Resources (equal); supervision (equal). **weidong Zhao:** Resources (equal); supervision (equal). **JIEQING ZHANG:** Resources (equal); supervision (equal). **Wenjun Cheng:** Resources (equal); supervision (equal). **Hongyan Guo:** Resources (equal); supervision (equal). **Ruixia Guo:** Resources (equal); supervision (equal). **Fengxia Xue:** Resources (equal); supervision (equal). **Xipeng Wang:** Resources (equal); supervision (equal). **Lili Han:** Resources (equal); supervision (equal). **Xiaomao Li:** Resources (equal); supervision (equal). **Ping Zhang:** Resources (equal); supervision (equal). **Jianguo Zhao:** Resources (equal); supervision (equal). **Wenting Li:** Data curation (equal); resources (equal); supervision (equal). **Yingyu Dou:** Data curation (equal); resources (equal); supervision (equal). **Zizhuo Wang:** Data curation (equal); resources (equal); supervision (equal). **Jingbo Liu:** Data curation (equal); resources (equal); supervision (equal). **Kezhen Li:** Conceptualization (equal); methodology (equal). **Gang Chen:** Conceptualization (equal); methodology (equal). **Chaoyang Sun:** Conceptualization (equal); methodology (equal). **Beibei Wang:** Conceptualization (equal); project administration (equal); supervision (equal). **Xing Sheng Yang:** Conceptualization (equal); project administration (equal); supervision (equal).

## FUNDING INFORMATION

This research received the specific grant from The Nature and Science Foundation of China (82073259).

## CONFLICT OF INTEREST STATEMENT

The authors certify that there is no conflict of interest with any financial organization regarding the material discussed in the manuscript.

7

## ETHICAL APPROVAL

All procedures performed in studies involving human participants were by the ethical standards of the institutional and/or national research committee and with the 1964 Helsinki declaration and its later amendments or comparable ethical standards. This study was exempted by Institutional Review Boards in all centers and complied with the Ethical Review Measures of Biomedical Research involving Human in China. The treatment of the enrolled women did not change along with the purpose of the study. No woman consent was needed because the data were analyzed anonymously in a de‐identified fashion, and it was a retrospective study.

## Supporting information


Data S1. Supplementary Materials.

Tables S1.–S7.

## Data Availability

The datasets used during the current study are available from the corresponding author on reasonable request.
