# Supplementary material for: It is not the time to abandon intraoperative frozen section in endometrioid adenocarcinoma: A large‐scale, multi‐center, and retrospective study
Source: Cancer Med. 2023 Jan 31;12(7):8897–910. doi: 10.1002/cam4.5643 (PMC10134352; doi:10.1002/cam4.5643)
Supplement: Supplementary file 1 — Data S1. Supplementary Materials. [file CAM4-12-8897-s002.docx]

Supplementary materials

**MRI**

1. Patients eligibility for inclusion was based on the following criteria: 1) preoperative MRI performed no longer than 6 weeks before surgery; 2) complete imaging protocol especially T2WI. MRI scans affected by artifacts were excluded.

2. All MRI images were re-evaluated specifically on myometrial invasion, extension, localization, and cervical extension by at least two radiologists with experience in pelvic MRI. At the time of re-check, the radiologist was aware of the diagnosis of endometrial carcinoma, but was blind to the results of surgery and the pathology report. Myometrial invasion was detected with MRI by the recognition of the disruption or discontinuity of the so-called ‘‘junctional zone’’ (JZ). According to the 1988 FIGO classification, myometrial invasion was considered as absent (non-invasive), ＜50% (superficial), and ≥50% (deep).

3. Myometrial invasion was evaluated according to previously published criteria(1, 2). Assessment of myometrial invasion was based both on T2WI and dynamic images. Cases showing poor defined tumor–myometrium interfaces on T2WI due to motion artifacts or lesions as intramural leiomyomas or adenomyosis were better assessed on dynamic sequences. On T2WI, a tumor limited to the endometrium was diagnosed when the JZ appeared to be intact. Myometrial invasion was diagnosed either by disruption or discontinuity of the JZ or by the extension of tumor signal intensity into the myometrium. When the JZ was not visible, a smooth endometrium–myometrium interface was considered as a no infiltrative tumor, while an irregular interface was considered as an infiltrative tumor. In the dynamic study, an intact and smooth sub endometrial enhancement was considered as a no infiltrative tumor. In contrast, disruption or irregularity of the sub endometrial enhancement was indicative of myometrial invasion. When the signal intensity of the tumor extended into the outer half of the myometrium, either on T2WI or dynamic images, deep myometrial invasion was diagnosed.

**CT**

1. Patients eligibility for inclusion was based on the following criteria: 1) preoperative CT performed no longer than 6 weeks before surgery; 2) complete imaging protocol. CT scans affected by artifacts were excluded.

2. CT images were re-interpreted by at least two radiologists in consensus. At the time of re-check, the radiologist was aware of the diagnosis of endometrial carcinoma, but was blind to the results of surgery and the pathology report.

3. The CT data for analysis included: (1) presence of tumor, detected either as a hypodense mass within the uterine cavity or as an area of endometrial thickening (measuring >5 mm in postmenopausal women); (2) assessment of presence and depth of myometrial infiltration, detected as an irregular interface between the tumor and the normally enhanced myometrium and classified as absent (noninvasive), superficial invasion, and deep invasion(2, 3). The CT criteria for interpreting the depth of myometrial invasion are illustrated as below. Noninvasive: Normally appearing endometrium or thickened endometrium with or without a mass, sharply demarcated from the normal myometrium. Superficial invasion: Mass, irregularly demarcated from the normally enhanced myometrium, invading <50% of the myometrial thickness. Deep invasion: Mass invading more than or equal to 50% of the myometrial thickness.

**Intraoperative gross examination**

1. Patients eligibility for inclusion was based on the following criteria: 1) complete gross examination protocol; 2) photograph of gross specimen.

2. Operation notes and gross photographs were re-check by at least two clinical pathologists. At the time of re-check, the pathologists were aware of the diagnosis of endometrial carcinoma, but was blind to the results of surgery and the pathology report.

3. After removal of the uterus, using scissors, the uterus was bivalved longitudinally through the cervical canal and the endometrial cavity. Any ulcerative, polypoid, poorly defined, hemorrhagic, necrotic, solid, or infiltrative mass(es) in the endometrial cavity are considered “suspicious for malignancy,”(4). The endometrial cavity was inspected and one or more transverse, full-thickness incisions were made through the tumor, myometrium, and serosa(5). Intraoperative estimation of gross myometrial invasion was made from the cut which appeared to show the deepest invasion and was classified as confined to endometrium, ＜50% or ≥50% of the myometrium (6). The grossly estimated depth of myometrial invasion was noted in the patient’s operative record.

**Intraoperative frozen section**

1. Patients eligibility for inclusion was based on the following criteria: 1) complete intraoperative frozen section protocol; 2) intraoperative frozen section reports.

2. Pathology reports and procedure were re-check by at least two clinical pathologists. At the time of re-check, the pathologists were aware of the diagnosis of endometrial carcinoma, but was blind to the results of surgery and the pathology report.

3. After a hysterectomy, the uterine anterior wall was sectioned through the cervical canal and the endometrial cavity longitudinally and then was sectioned horizontally from the fundus to the ostium of the fallopian tube. The endometrial cavity was carefully inspected. Then, the endo-myometrium was sliced transversely at 4–5 mm intervals, and the cut surfaces were subjected to gross examination. Clinical pathologists assessed the deepest point of invasion and oversaw the selection of sections for freezing. One full-thickness section of an endometrial tumor, grossly assessed to be deepest point of invasion, with underlying myometrium and serosa, was frozen in optimal cutting temperature media and sectioned on a cryostat into 5-um slices. Three different levels were cut, separated by approximately 25 to 50 ums, which were then taken, mounted on a glass microscopic slide, rapidly stained with hematoxylin and eosin, dehydrated, and cover-slipped. The slices were mounted on glass slides stained with hematoxylin and eosin, and evaluated microscopically for histologic type of tumor, FIGO histologic grade, and extent of myometrial invasion. Occasionally, an additional section was frozen when the first slides showed equivocal findings or the sections were of particularly poor quality. The microscopic diagnosis was performed by two pathologists. In most cases, the frozen section remnant was then submitted for permanent histology, and several additional sections of tumor were paraffin-embedded and used for the final microscopic analysis. Frozen section estimation of myometrial invasion and histological grades was made from the cut which show the deepest invasion.

**Paraffin section**

1. Preoperative histological examination of the endometrial tumor was performed by diagnostic curettage or hysteroscopy.

2. We consider paraffin section pathology after hysterectomy as the gold standard for the analyses of myometrial invasion and histological grades.

3. Final pathologic evaluation of the paraffin specimens were performed as standard procedure. All the definitive pathologic evaluations have been reviewed by at least two reference pathologists.

4. In our study, the results of myometrial invasion appealed to the FIGO 1988, including no myometrial invasion, superficial myometrial invasion, and deep myometrial invasion. No myometrial invasion was defined as that microscopic lesions are confined to the endometrium. Superficial myometrial invasion was defined as that the depth of lesion involvements are less than 1/2 of myometrium, microscopically. Meanwhile, deep myometrial invasion was defined as that the depth of lesion involvements are greater than or equal to 1/2 of myometrium. For the assessment of myometrial invasion, account needs to be taken of the endo-myometrial junction which is undulating and multifocal features of endometrial lesions. If endometrial tumor is obvious, 2-4 pieces should be taken from the deepest part of tumor invasion (including tumor surface to serous layer). If there is no obvious tumor in the hysterectomy specimen, the endometrium and its adjacent superficial muscle layer should be taken entirely. Rare infiltration patterns such as microcystic, elongated, fragmentary (MELF) infiltration, malignant adenomatous infiltration, and single cell infiltration should be noted.

5. Referring to the latest WHO classification of endometrial cancer, the histological types of endometrial cancer include: endometrioid adenocarcinoma, mucinous carcinoma, serous, clear cell, undifferentiated carcinoma, dedifferentiated carcinoma, mixed carcinoma, neuroendocrine tumor and carcinosarcoma, etc. There are three subtypes of endometrioid adenocarcinoma: squamous differentiation type, villous gland tubular type and secretory type. According to the proportion of glandular structure and solid area (except squamous metaplasia) and the atypia of the nucleus of glandular epithelium, there are three grades: G1 with solid structure ≤5% in adenocarcinoma, G2 with solid structure accounts for 6%-50%, and G3 with solid structure >50%. If more than 50% of the tumor nuclei are high-grade, upgrading to one level higher. The International Society of Gynecological Pathology (ISGyP) recommends that endometrioid adenocarcinoma be classified into low-grade (G1, G2) and high-grade (G3).

Reference

1. Sironi S, Taccagni G, Garancini P, Belloni C, DelMaschio A. Myometrial invasion by endometrial carcinoma: assessment by MR imaging. AJR American journal of roentgenology. 1992;158(3):565-9.

2. Lee JH, Dubinsky T, Andreotti RF, Cardenes HR, Dejesus Allison SO, Gaffney DK, et al. ACR appropriateness Criteria® pretreatment evaluation and follow-up of endometrial cancer of the uterus. Ultrasound quarterly. 2011;27(2):139-45.

3. Hardesty LA, Sumkin JH, Hakim C, Johns C, Nath M. The ability of helical CT to preoperatively stage endometrial carcinoma. AJR American journal of roentgenology. 2001;176(3):603-6.

4. Desouki MM, Li Z, Hameed O, Fadare O. Intraoperative Pathologic Consultation on Hysterectomy Specimens for Endometrial Cancer: An Assessment of the Accuracy of Frozen Sections, "Gross-Only" Evaluations, and Obtaining Random Sections of a Grossly "Normal" Endometrium. Am J Clin Pathol. 2017;148(4):345-53.

5. Smith TM, Smith SC, Delancey JO, Fenner DE, Schimpf MO, Roh MH, et al. Pathologic evaluation of explanted vaginal mesh: interdisciplinary experience from a referral center. Female pelvic medicine & reconstructive surgery. 2013;19(4):238-41.

6. Franchi M, Ghezzi F, Melpignano M, Cherchi PL, Scarabelli C, Apolloni C, et al. Clinical value of intraoperative gross examination in endometrial cancer. Gynecol Oncol. 2000;76(3):357-61.
