# Supplementary material for: It is not the time to abandon intraoperative frozen section in endometrioid adenocarcinoma: A large‐scale, multi‐center, and retrospective study
Source: Cancer Med. 2023 Jan 31;12(7):8897–910. doi: 10.1002/cam4.5643 (PMC10134352; doi:10.1002/cam4.5643)
Supplement: Supplementary file 2 — Tables S1.–S7. [file CAM4-12-8897-s001.docx]

Supplementary Table 1 Statistical analysis

| Statistical method | Categorical measurements are summarized as percentages using cross-tables. |
| --- | --- |
| Sensitivity | True positive rate, TPR=TP/ (TP+ FN) |
| Specificity | True negative rate, TNR=TN/ (FP + TN) |
| PPV | Positive predictive value=TP/ (TP+FP) |
| NPV | Negative predictive value=TN/ (TN+FN) |
| Accuracy | TP+TN/ (TP+FN+FP+TN) |
| Mc-Nemar test | Difference between sensitivity and specificity in categorical variables |
| Cohen’s Kappa | Consistency; unordered dichotomous variables (< 0, consistency less than chance; < 0.20, slight consistency; 0.21-0.40, fair consistency; 0.41-0.60, moderate consistency; 0.61-0.80, substantial consistency; > 0.8, almost perfect consistency) |
| Cronbach’s α-inter  rate correlation | Consistency; ordered classified variables (> 0.90, high correlation; 0.8-0.9, acceptable correlation; 0.70-0.8, scale needs amending; < 0.7, discard) |
| Composite diagnosis  in parallel mode | All related tests are performed on patients, and the final positive identification of parallel mode is determined when any test is positive. This combination mode will decrease diagnostic specificity and improve sensitivity. |
| Bivariate logistic  regression | Evaluates independent risk factors for dichotomous outcomes. The Hosmer–Lemeshow goodness-of-fit χ2 test was used to assess the model fit. The model's overall significance was assessed by the -2 loglikelihood ratio. |
| Odds ratios | Odds ratios (ORs) from logistic regression are measures of association between an exposure and an outcome. The ORs represent the odds that an outcome will occur given a particular exposure, compared to the odds of the outcome occurring in the absence of this exposure. |

Footnote TP: true positive; TN: true negative; FP: false positive; FN: false negative.

Supplementary Table 2 Grouping data by year of diagnosis.

| Group | Central registries  N= | Year of diagnosis |
| --- | --- | --- |
| 2000-2008 | 38 | 2000 |
|  | 41 | 2001 |
|  | 35 | 2002 |
|  | 36 | 2003 |
|  | 50 | 2004 |
|  | 82 | 2005 |
|  | 120 | 2006 |
|  | 203 | 2007 |
|  | 205 | 2008 |
| 2009-2010 | 286 | 2009 |
|  | 394 | 2010 |
| 2011-2012 | 570 | 2011 |
|  | 628 | 2012 |
| 2013-2014 | 762 | 2013 |
|  | 986 | 2014 |
| 2015-2016 | 1519 | 2015 |
|  | 1564 | 2016 |
| 2017-2019 | 2061 | 2017 |
|  | 278 | 2018 |
|  | 127 | 2019 |
| Total | 9985 |  |

Supplementary Table 3 Stratified comparisons of utility in detecting deep myometrial invasion based on the diagnosis year.

| MI | | | Postoperative PS | | |  | Postoperative PS | | |  | Postoperative PS | | |  | Postoperative PS | | |
| --- | --- | --- | --- | --- | --- | --- | --- | --- | --- | --- | --- | --- | --- | --- | --- | --- | --- |
|  |  |  | No/<50% | ≥50% | Total |  | No/<50% | ≥50% | Total |  | No/<50% | ≥50% | Total |  | No/<50% | ≥50% | Total |
| Total | No/<50% | CT | 827 | 139 | 966 | MRI | 2725 | 351 | 3076 | IGE | 5323 | 577 | 5900 | IFS | 1951 | 90 | 2041 |
|  | ≥50% |  | 53 | 41 | 94 |  | 363 | 240 | 603 |  | 617 | 683 | 1300 |  | 24 | 238 | 262 |
|  | Total |  | 880 | 180 | 1060 |  | 3088 | 591 | 3679 |  | 5940 | 1260 | 7200 |  | 1975 | 328 | 2303 |
| 2000-2008 | No/<50% |  | 33 | 5 | 38 |  | 53 | 1 | 54 |  | 513 | 55 | 568 |  | 96 | 4 | 100 |
|  | ≥50% |  | 3 | 4 | 7 |  | 15 | 6 | 21 |  | 67 | 68 | 135 |  | 0 | 15 | 15 |
|  | Total |  | 36 | 9 | 45 |  | 68 | 7 | 75 |  | 580 | 123 | 703 |  | 96 | 19 | 115 |
| 2009-2010 | No/<50% |  | 106 | 21 | 127 |  | 91 | 6 | 97 |  | 417 | 50 | 467 |  | 117 | 6 | 123 |
|  | ≥50% |  | 5 | 6 | 11 |  | 17 | 13 | 30 |  | 38 | 55 | 93 |  | 1 | 14 | 15 |
|  | Total |  | 111 | 27 | 138 |  | 108 | 19 | 127 |  | 455 | 105 | 560 |  | 118 | 20 | 138 |
| 2011-2012 | No/<50% |  | 97 | 22 | 119 |  | 185 | 32 | 217 |  | 765 | 65 | 830 |  | 259 | 9 | 268 |
|  | ≥50% |  | 7 | 11 | 18 |  | 50 | 10 | 60 |  | 76 | 87 | 163 |  | 4 | 28 | 32 |
|  | Total |  | 104 | 33 | 137 |  | 235 | 42 | 277 |  | 841 | 152 | 993 |  | 263 | 37 | 300 |
| 2013-2014 | No/<50% |  | 134 | 26 | 160 |  | 497 | 47 | 544 |  | 986 | 101 | 1087 |  | 267 | 15 | 282 |
|  | ≥50% |  | 16 | 4 | 20 |  | 68 | 48 | 116 |  | 119 | 121 | 240 |  | 4 | 36 | 40 |
|  | Total |  | 150 | 30 | 180 |  | 565 | 95 | 660 |  | 1105 | 222 | 1327 |  | 271 | 51 | 322 |
| 2015-2016 | No/<50% |  | 292 | 43 | 335 |  | 1105 | 156 | 1261 |  | 1471 | 158 | 1629 |  | 686 | 26 | 712 |
|  | ≥50% |  | 14 | 8 | 22 |  | 131 | 78 | 209 |  | 184 | 174 | 358 |  | 9 | 77 | 86 |
|  | Total |  | 306 | 51 | 357 |  | 1236 | 234 | 1470 |  | 1655 | 332 | 1987 |  | 695 | 103 | 798 |
| 2017-2019 | No/<50% |  | 165 | 22 | 187 |  | 794 | 109 | 903 |  | 1171 | 148 | 1319 |  | 526 | 30 | 556 |
|  | ≥50% |  | 8 | 8 | 16 |  | 82 | 85 | 167 |  | 133 | 178 | 311 |  | 6 | 68 | 74 |
|  | Total |  | 173 | 30 | 203 |  | 876 | 194 | 1070 |  | 1304 | 326 | 1630 |  | 532 | 98 | 630 |

Supplementary Table 4 Grouping data by medical centers.

| Group | Central registries  N= | Online web-based database |
| --- | --- | --- |
| 1 | 947 | Obstetrics and Gynecology Hospital of Fudan University |
|  | 25 | Union Hospital affiliated to Huazhong University of Science and Technology |
|  | 30 | Ningxia Medical University General Hospital |
|  | 12 | International Peace Maternal and Child Health Hospital |
|  | 12 | Shanghai First Maternal and Infant Health Hospital |
|  | 25 | Jiangsu Province Hospital |
|  | 35 | West China the Second Hospital |
|  | 40 | Urumqi Autonomous Region People's Hospital |
|  | 54 | Xiangya Hospital Central South University |
|  | 65 | Maternal and Child Health Hospital of Shenzhen City |
|  | 96 | Tianjin Central Obstetrics and Gynecology Hospital |
|  | 136 | Second Hospital of Shandong University |
|  | 137 | Xiangya III Hospital of Central South University |
|  | 184 | Xinhua Hospital affiliated to Shanghai Jiao tong University |
|  | 186 | The Third Affiliated Hospital of Sun Yat-sen University |
| 2 | 901 | Qilu Hospital of Shandong University |
|  | 785 | Yantai Yuhuangding Hospital |
|  | 750 | Cancer Hospital affiliated to Harbin Medical University |
| 3 | 868 | Obstetrics and Gynecology Hospital affiliated to Zhejiang University School of Medicine |
|  | 794 | Sun Yat-sen University Cancer Center |
| 4 | 814 | The First Affiliated Hospital of Sun Yat-sen University |
|  | 523 | Peking University People's Hospital |
|  | 528 | Affiliated Tumor Hospital of Guangxi Medical University |
| 5 | 207 | Shengjing Hospital |
|  | 208 | Affiliated Hospital of Qingdao Medical College |
|  | 274 | Tongji Hospital affiliated to Huazhong University of Science and Technology |
|  | 278 | Tianjin Medical University General Hospital |
|  | 281 | The First Affiliated Hospital of Zhengzhou University |
|  | 362 | Peking University Third Hospital, |
|  | 428 | The First Affiliated Hospital of University of Science and Technology of China |
| Total | 9985 |  |

Supplementary Table 5 Stratified comparisons of utility in detecting deep myometrial invasion based on the medical centers.

| MI | | | Postoperative PS | | |  | Postoperative PS | | |  | Postoperative PS | | | |  | | Postoperative PS | | | |
| --- | --- | --- | --- | --- | --- | --- | --- | --- | --- | --- | --- | --- | --- | --- | --- | --- | --- | --- | --- | --- |
|  |  |  | No/<50% | ≥50% | Total |  | No/<50% | ≥50% | Total |  | No/<50% | ≥50% | Total |  | | No/<50% | | ≥50% | Total |  |
| Total | No/<50% | CT | 827 | 139 | 966 | MRI | 2725 | 351 | 3076 | IGE | 5323 | 577 | 5900 | IFS | | 1951 | | 90 | 2041 |  |
|  | ≥50% |  | 53 | 41 | 94 |  | 363 | 240 | 603 |  | 617 | 683 | 1300 |  |  | 24 | | 238 | 262 |  |
|  | Total |  | 880 | 180 | 1060 |  | 3088 | 591 | 3679 |  | 5940 | 1260 | 7200 |  |  | 1975 | | 328 | 2303 |  |
| 1 | No/<50% |  | 78 | 8 | 86 |  | 827 | 114 | 941 |  | 465 | 73 | 538 |  |  | 917 | | 51 | 968 |  |
|  | ≥50% |  | 2 | 2 | 4 |  | 77 | 49 | 126 |  | 45 | 55 | 100 |  |  | 5 | | 124 | 129 |  |
|  | Total |  | 80 | 10 | 90 |  | 904 | 163 | 1067 |  | 510 | 128 | 638 |  |  | 922 | | 175 | 1097 |  |
| 2 | No/<50% |  | 92 | 17 | 109 |  | 651 | 83 | 734 |  | 1313 | 141 | 1454 |  |  | 509 | | 19 | 528 |  |
|  | ≥50% |  | 3 | 2 | 5 |  | 77 | 63 | 140 |  | 154 | 151 | 305 |  |  | 3 | | 44 | 47 |  |
|  | Total |  | 95 | 19 | 114 |  | 728 | 146 | 874 |  | 1467 | 292 | 1759 |  |  | 512 | | 63 | 575 |  |
| 3 | No/<50% |  | 355 | 59 | 414 |  | 480 | 71 | 551 |  | 1023 | 85 | 1108 |  |  | 60 | | 1 | 61 |  |
|  | ≥50% |  | 15 | 16 | 31 |  | 26 | 19 | 45 |  | 126 | 143 | 269 |  |  | 1 | | 3 | 4 |  |
|  | Total |  | 370 | 75 | 445 |  | 506 | 90 | 596 |  | 1149 | 228 | 1377 |  |  | 61 | | 4 | 65 |  |
| 4 | No/<50% |  | 275 | 52 | 327 |  | 424 | 40 | 464 |  | 1237 | 137 | 1374 |  |  | 83 | | 3 | 86 |  |
|  | ≥50% |  | 31 | 19 | 50 |  | 109 | 60 | 169 |  | 159 | 175 | 334 |  |  | 2 | | 9 | 11 |  |
|  | Total |  | 306 | 71 | 377 |  | 533 | 100 | 633 |  | 1396 | 312 | 1708 |  |  | 85 | | 12 | 97 |  |
| 5 | No/<50% |  | 27 | 3 | 30 |  | 343 | 43 | 386 |  | 1285 | 141 | 1426 |  |  | 382 | | 16 | 398 |  |
|  | ≥50% |  | 2 | 2 | 4 |  | 74 | 49 | 123 |  | 133 | 159 | 292 |  |  | 13 | | 58 | 71 |  |
|  | Total |  | 29 | 5 | 34 |  | 417 | 92 | 509 |  | 1418 | 300 | 1718 |  |  | 395 | | 74 | 469 |  |

Supplementary Table 6 Stratified comparisons of utility in detecting high-grade based on the diagnosis year.

| High-grade EA | | | Postoperative PS | | |  | Postoperative PS | | |  | Postoperative PS | | |
| --- | --- | --- | --- | --- | --- | --- | --- | --- | --- | --- | --- | --- | --- |
|  |  |  | No | Yes | Total |  | No | Yes | Total |  | No | Yes | Total |
| Total | No | D&C | 6809 | 792 | 7601 | Hys | 2031 | 255 | 2286 | IFS | 2675 | 199 | 2874 |
|  | Yes |  | 161 | 286 | 447 |  | 23 | 41 | 64 |  | 81 | 180 | 261 |
|  | Total |  | 6970 | 1078 | 8048 |  | 2054 | 296 | 2350 |  | 2756 | 379 | 3135 |
| 2000-2008 | No |  | 590 | 77 | 667 |  | 126 | 20 | 146 |  | 142 | 8 | 150 |
|  | Yes |  | 15 | 25 | 40 |  | 1 | 0 | 1 |  | 0 | 10 | 10 |
|  | Total |  | 605 | 102 | 707 |  | 127 | 20 | 147 |  | 142 | 18 | 160 |
| 2009-2010 | No |  | 472 | 59 | 531 |  | 125 | 17 | 142 |  | 160 | 9 | 169 |
|  | Yes |  | 10 | 26 | 36 |  | 2 | 2 | 4 |  | 2 | 12 | 14 |
|  | Total |  | 482 | 85 | 567 |  | 127 | 19 | 146 |  | 162 | 21 | 183 |
| 2011-2012 | No |  | 800 | 89 | 889 |  | 187 | 23 | 210 |  | 365 | 12 | 377 |
|  | Yes |  | 17 | 42 | 59 |  | 3 | 1 | 4 |  | 1 | 25 | 26 |
|  | Total |  | 817 | 131 | 948 |  | 190 | 24 | 214 |  | 366 | 37 | 403 |
| 2013-2014 | No |  | 1270 | 134 | 1404 |  | 329 | 35 | 364 |  | 389 | 31 | 420 |
|  | Yes |  | 32 | 49 | 81 |  | 5 | 4 | 9 |  | 31 | 22 | 53 |
|  | Total |  | 1302 | 183 | 1485 |  | 334 | 39 | 373 |  | 420 | 53 | 473 |
| 2015-2016 | No |  | 2132 | 265 | 2397 |  | 626 | 87 | 713 |  | 885 | 72 | 957 |
|  | Yes |  | 46 | 77 | 123 |  | 6 | 18 | 24 |  | 40 | 65 | 105 |
|  | Total |  | 2178 | 342 | 2520 |  | 632 | 105 | 737 |  | 925 | 137 | 1062 |
| 2017-2019 | No |  | 1545 | 168 | 1713 |  | 638 | 73 | 711 |  | 734 | 67 | 801 |
|  | Yes |  | 41 | 67 | 108 |  | 6 | 16 | 22 |  | 7 | 46 | 53 |
|  | Total |  | 1586 | 235 | 1821 |  | 644 | 89 | 733 |  | 741 | 113 | 854 |

Supplementary Table 7 Stratified comparisons of utility in detecting high-grade based on the medical centers.

| High-grade EA | | | Postoperative PS | | |  | Postoperative PS | | |  | Postoperative PS | | | |
| --- | --- | --- | --- | --- | --- | --- | --- | --- | --- | --- | --- | --- | --- | --- |
|  |  |  | No | Yes | Total |  | No | Yes | Total |  | No | Yes | Total |  |
| Total | No | D&C | 6809 | 792 | 7601 | Hys | 2031 | 255 | 2286 | IFS | 2675 | 199 | 2874 |  |
|  | Yes |  | 161 | 286 | 447 |  | 23 | 41 | 64 |  | 81 | 180 | 261 |  |
|  | Total |  | 6970 | 1078 | 8048 |  | 2054 | 296 | 2350 |  | 2756 | 379 | 3135 |  |
| 1 | No |  | 1413 | 168 | 1581 |  | 651 | 87 | 738 |  | 1066 | 89 | 1155 |  |
|  | Yes |  | 37 | 58 | 95 |  | 11 | 18 | 29 |  | 68 | 99 | 167 |  |
|  | Total |  | 1450 | 226 | 1676 |  | 662 | 105 | 767 |  | 1134 | 188 | 1322 |  |
| 2 | No |  | 1662 | 166 | 1828 |  | 513 | 58 | 571 |  | 715 | 49 | 764 |  |
|  | Yes |  | 28 | 45 | 73 |  | 4 | 6 | 10 |  | 5 | 41 | 46 |  |
|  | Total |  | 1690 | 211 | 1901 |  | 517 | 64 | 581 |  | 720 | 90 | 810 |  |
| 3 | No |  | 984 | 165 | 1149 |  | 206 | 29 | 235 |  | 99 | 12 | 111 |  |
|  | Yes |  | 34 | 82 | 116 |  | 2 | 7 | 9 |  | 2 | 7 | 9 |  |
|  | Total |  | 1018 | 247 | 1265 |  | 208 | 36 | 244 |  | 101 | 19 | 120 |  |
| 4 | No |  | 1401 | 156 | 1557 |  | 302 | 33 | 335 |  | 171 | 10 | 181 |  |
|  | Yes |  | 38 | 70 | 108 |  | 3 | 4 | 7 |  | 1 | 10 | 11 |  |
|  | Total |  | 1439 | 226 | 1665 |  | 305 | 37 | 342 |  | 172 | 20 | 192 |  |
| 5 | No |  | 1349 | 137 | 1486 |  | 359 | 48 | 407 |  | 624 | 39 | 663 |  |
|  | Yes |  | 24 | 31 | 55 |  | 3 | 6 | 9 |  | 5 | 23 | 28 |  |
|  | Total |  | 1373 | 168 | 1541 |  | 362 | 54 | 416 |  | 629 | 62 | 691 |  |
